# Microbiota-based interventions for autism spectrum disorder: a systematic review of efficacy and clinical potential

**DOI:** 10.3389/fmicb.2025.1648118

**Published:** 2025-09-26

**Authors:** Hana Taha, Ahmad Issa, Zaid Muhanna, Majdi Al-Shehab, Tuleen Wadi, Suhib Awamleh, Yousef A. Ateiwi, Mohammad Abusido, Vanja Berggren

**Affiliations:** ^1^Department of Family and Community Medicine, School of Medicine, The University of Jordan, Amman, Jordan; ^2^Department of Neurobiology, Care Science and Society, Karolinska Institutet, Stockholm, Sweden

**Keywords:** autism spectrum disorder, probiotics, prebiotics, synbiotics, fecal microbiota, transplantation, microbiota-based therapies

## Abstract

**Purpose:**

Autism spectrum disorder (ASD) is increasingly linked to gut microbiota imbalances, influencing both behavioral and gastrointestinal (GI) symptoms. This systematic review assesses the efficacy of microbiota-based interventions, including probiotics, prebiotics, synbiotics, and fecal microbiota transplantation (FMT), in improving ASD-related symptoms, aiming to provide insights into their therapeutic potential and inform future clinical applications.

**Methods:**

A comprehensive systematic review was conducted following PRISMA guidelines and registered in PROSPERO (CRD42024615043). A structured literature search was performed in PubMed, Cochrane Library, and Scopus to identify peer-reviewed English-language studies. Eligible studies included randomized controlled trials (RCTs), non-randomized trials (NRTs), and retrospective studies assessing the impact of microbiota-based interventions on ASD-related behavioral and GI outcomes. Two independent reviewers conducted study selection, data extraction, and quality assessment using standardized risk-of-bias tools.

**Results:**

33 studies were included, consisting of 16 RCTs, 14 NRTs, and 3 retrospective studies. Among them, 15 assessed probiotics, 4 prebiotics, 5 synbiotics, and 9 FMT. Probiotics showed moderate behavioral improvements in ASD, with multi-strain formulations being more effective than single strains. Prebiotics and synbiotics yielded mixed results, with some studies indicating benefits in behavioral and GI symptoms. FMT demonstrated the most consistent and sustained improvements in both ASD-related behaviors and GI function. Adverse events were minimal, primarily involving transient GI symptoms.

**Conclusion:**

Microbiota-targeted interventions, particularly FMT, hold promise for managing ASD symptoms, though probiotics, prebiotics, and synbiotics present variable efficacy. Standardized protocols, larger controlled trials, and personalized microbiome-based approaches are necessary to refine these therapeutic strategies and enhance clinical applicability.

**Systematic review registration:**

https://www.crd.york.ac.uk/PROSPERO/view/CRD42024615043, identifier CRD42024615043.

## Introduction

Autism spectrum disorder (ASD) is a group of neurodevelopmental conditions characterized by repetitive behaviors, restricted interests, and varying degrees of difficulty with social interaction and communication (American Psychiatric Association, 2013). Symptoms typically emerge early in life and present with diverse patterns and severity. Recent estimates suggest that ASD affects approximately 1 in 36 children in the United States, reflecting a steady rise in diagnoses over the past few decades (Maenner et al., 2021). While genetic and environmental factors contribute to its development, emerging research highlights the role of systemic physiological disturbances, particularly gastrointestinal (GI) dysfunction and gut microbiota imbalances, in ASD pathophysiology (Sauer et al., 2021).

In addition to core behavioral symptoms, up to 70 percent of individuals with ASD experience GI issues, including chronic constipation, diarrhea, abdominal pain, and gastroesophageal reflux (Valicenti-McDermott et al., 2006). Studies suggest a strong link between GI symptoms and ASD severity, indicating a bidirectional relationship between gut health and neurological outcomes (Ferguson et al., 2019; Mazefsky et al., 2014).

The gut-brain axis mediates this connection, involving complex interactions between the central nervous system, gut microbiota, immune responses, and endocrine signaling (Cryan et al., 2019). Disruptions in this axis, such as increased intestinal permeability, systemic inflammation, and gut dysbiosis, have been implicated in ASD (Sharon et al., 2019). Preclinical studies further suggest that microbiota-derived metabolites, such as short-chain fatty acids, influence neuroinflammation and synaptic plasticity, supporting the rationale for microbiota-targeted therapies (Fattorusso et al., 2019).

Microbiota-targeted therapies, including probiotics, prebiotics, synbiotics, and fecal microbiota transplantation (FMT), have gained attention as potential interventions for ASD-related, GI, and behavioral symptoms. Probiotics are live microorganisms that confer health benefits when administered in adequate amounts (Hill et al., 2014). While prebiotics are non-digestible substrates that act as nutrients for beneficial host-associated microorganisms, including both administered probiotic strains and resident microbes, with common examples being inulin, fructo-oligosaccharides (FOS), and galacto-oligosaccharides (GOS) (Hill et al., 2014; Davani-Davari et al., 2019). Multiple studies have reported improvements in ASD-related outcomes following their use (Raghavan et al., 2022; Inoue et al., 2019; Grimaldi et al., 2018). Synbiotics combine both elements to enhance microbial colonization and metabolic activity (Svedlund et al., 1988). Preliminary studies suggest that probiotics, particularly strains of *Bifidobacterium*, may reduce GI distress and moderately improve behavioral symptoms in ASD (Sanctuary et al., 2019). FMT, which involves restoring microbial diversity by transferring processed stool from a healthy donor, has demonstrated promising results, including improvements in both GI symptoms and social responsiveness (Dossaji et al., 2023).

This review synthesizes contemporary evidence on probiotics, prebiotics, synbiotics, and FMT as therapeutic strategies for addressing ASD-related behavioral and GI symptoms. We evaluate their safety, clinical efficacy, and underlying molecular mechanisms. Through a critical review of the current literature, we aim to provide an overview of the effectiveness and tolerability of these therapies and offer insights to guide future research in ASD management.

## Methods

### Protocol and registration

This systematic review was conducted following the Preferred Reporting Items for Systematic Reviews and Meta-Analyses (PRISMA) statement and registered in the International Prospective Register of Systematic Reviews (PROSPERO CRD42024615043).

### Literature search

We conducted a comprehensive literature search across PubMed, Cochrane Library, and Scopus to identify full-text English articles published from inception to November 16, 2024. An updated search was performed on February 19, 2025. Our search strategy incorporated a combination of free-text keywords and MeSH terms related to ASD and gut microbiota interventions. The full search strategy is provided in Supplementary Table S1.

In our search strategy, we placed no restrictions on publication date or country of origin. Additionally, two independent reviewers screened the reference lists of the retrieved literature to identify any potentially eligible studies that may have been missed.

### Study selection

We included studies that investigated FMT, prebiotics, probiotics, or synbiotics in patients with ASD. To be eligible, studies had to assess behavioral or GI symptoms using validated measures both before and after the intervention. Studies that only analyzed microbiome changes without evaluating symptom outcomes were excluded. We also excluded studies that did not contain original data, such as meta-analyses, reviews, study protocols, conference abstracts, and letters to the editor. Additional exclusions included case reports, case series, non-English publications, and animal studies.

We utilized Rayyan software to facilitate the screening process (Ouzzani et al., 2016). After uploading the titles and abstracts from the preliminary search and removing duplicates, two independent reviewers screened the titles and abstracts based on the predefined inclusion and exclusion criteria. Subsequently, two other independent reviewers assessed the full-text articles of studies that passed the initial screening. Any disagreements were resolved through consensus between the reviewers.

### Study outcomes and score interpretation

The primary focus of this review was to assess changes in ASD symptoms, including behavioral symptoms, social interactions, repetitive behaviors, and communication abilities. These outcomes were measured using validated rating scales and assessment tools such as the Autism Treatment Evaluation Checklist (ATEC), the Childhood Autism Rating Scale (CARS), the Clinical Global Impression–Severity (CGI-S), the Aberrant Behavior Checklist (ABC), the Autism Behavior Checklist (AuBC), and the Social Responsiveness Scale (SRS).

Secondary outcomes included changes in GI symptoms, such as stool consistency, frequency, abdominal discomfort, and overall GI health. These were assessed using tools like the GI Severity Index (GSI) and the PedsQL GI Symptoms Scales, along with other qualitative measures.

For impairment-type scales (e.g., CARS, SRS-2, Aberrant Behavior Checklist, ATEC, GSRS, and GSI/6-GSI), lower scores indicate improvement; for functional scales (e.g., Vineland Adaptive Behavior Scales), higher scores indicate improvement. We explicitly name the instrument at first mention (e.g., Aberrant Behavior Checklist vs. Autism Behavior Checklist) and use consistent acronyms thereafter to avoid ambiguity. The scales used in included studies are summarized in Table 1.

**Table 1:** Characteristics of scales and questionnaires applied across studies.

Instrument type (abbreviation)	Key domains	Rater	Scoring	Direction of improvement
Adaptive Behavior Assessment System–2 (ABAS-2)	Conceptual, Social, Practical adaptive skills (communication, self-care, social, community use, health & safety, academics, motor)	Parent, Teacher, Caregiver, or Self-report	Raw → Standard scores (composite indexes, percentiles)	Higher scores = better adaptive functioning
Aberrant Behavior Checklist (ABC)	Irritability, Lethargy/Social Withdrawal, Stereotypy, Hyperactivity/Noncompliance, Inappropriate Speech	Parent/caregiver	58 items (each 0–3), Total 0–174	Lower scores = fewer problem behaviors
ADOS Calibrated Severity Score (ADOS-CSS)	Autism symptom severity	Clinician	1–10	Lower scores = less severe autism symptoms
Achenbach System of Empirically Based Assessment (ASEBA)	Internalizing, Externalizing, Syndrome scales	Parent/Teacher	T-scores	Lower scores = fewer behavioral problems
Autism Behavior Checklist (AuBC)	Sensory, Relating, Body/Object Use, Language, Social/Self-Help	Parent/caregiver	57 items; weighted	Lower scores = fewer autism-related behaviors
Autism Treatment Evaluation Checklist (ATEC)	Speech/Language/Communication; Sociability; Sensory/Cognitive Awareness; Health/Physical/Behavior	Parent/caregiver	77 items; Total 0–179	Lower scores = fewer autism-related problems
Bristol Stool Form Scale (BSFS)	Stool form	Parent	Types 1–7	Scores toward Type 4 = healthier stool consistency
Childhood Autism Rating Scale (CARS/CARS-2)	Overall autism severity; social-communication + RRB	Clinician observation	15 items, 1–4 (½ steps); Total 15–60	Lower scores = less severe autism symptoms
Clinical Global Impression – Improvement (CGI-I)	Global improvement	Clinician	1–7	Lower scores = greater improvement
Clinical Global Impression – Severity (CGI-S)	Global severity	Clinician	1–7	Lower scores = less severe illness
GI Severity Index (GSI / 6-GSI)	Composite GI symptom severity	Parent	Version-dependent	Lower scores = fewer GI symptoms
GI Symptom Rating Scale (GSRS)	Reflux, Abdominal pain, Indigestion, Diarrhea, Constipation	Self/Parent proxy	15 items, 1–7	Lower scores = fewer GI symptoms
PedsQL™ GI Module	GI-related HRQoL	Parent/Child	0–100 (transformed)	Higher scores = better GI-related quality of life
Psychoeducational Profile–3 (PEP-3)	Communication, Motor, Maladaptive behaviors, Cognitive, Personal–self-care	Clinician/Examiner	Item ratings → Developmental age equivalents and percentiles	Higher scores = better developmental performance
Parent Global Impressions – III (PGI-III)	Global parent-rated severity and improvement	Parent	7-point Likert: Severity (PGI-S) and Improvement (PGI-I)	Lower PGI-S scores = less severe; Lower PGI-I scores = greater improvement
Parenting Stress Index (PSI)	Parent (competence, depression, attachment), Child (mood, adaptability, distractibility), Life stress	Parent (self-report)	Raw → Percentiles, Cutoffs	Lower scores = less parenting stress
Sleep Disturbance Scale for Children (SDSC)	Sleep initiation/maintenance, Breathing, Arousal, Sleep–wake transitions, Somnolence, Hyperhidrosis	Parent (proxy)	26 items, Likert 1–5 → Subscale + Total scores	Lower scores = fewer sleep disturbances
Social Responsiveness Scale-2 (SRS-2)	Social awareness, cognition, communication, motivation, RRB	Parent/Teacher	65 items → T-scores	Lower scores = fewer autism-related social impairments
Vineland Adaptive Behavior Scales (VABS-II/III)	Communication, Daily Living, Socialization, Motor; Adaptive Composite	Caregiver interview	Standard scores (M = 100, SD = 15)	Higher scores = better adaptive functioning

GI, Gastrointestinal; RRB, Restricted and Repetitive Behaviors; HRQoL, Health-Related Quality of Life.

### Data extraction

We developed a comprehensive data extraction form, which was validated by an expert to ensure the inclusion of all clinically relevant information. The extracted data included study design and setting, population characteristics, type of intervention and comparator, baseline measures, and reported outcomes. For qualitative synthesis, we specifically extracted data about the behavioral outcomes (e.g., social interaction, communication abilities, repetitive behaviors) and GI outcomes (e.g., stool consistency, frequency, abdominal discomfort, and overall GI health). To ensure accuracy, two independent reviewers performed data extraction, with discrepancies resolved through consultation with a third reviewer. Due to the high heterogeneity in interventions and reported outcomes across studies, a meta-analysis was not feasible. Instead, the findings were synthesized qualitatively.

### Quality assessment

The methodological quality of each included study was independently assessed by two reviewers, with disagreements resolved through discussion. Due to the heterogeneity in study designs, we employed multiple quality assessment tools. For randomized controlled trials (RCTs), we used the Revised Cochrane Risk-of-Bias Tool (RoB 2) (Sterne et al., 2019). For observational studies, we used a modified version of the Newcastle-Ottawa Scale (NOS) (Wells et al., 2000), as none of the studies included a control group. For interventional studies, we applied the Methodological Index for Non-Randomized Studies (MINORS), using the full version for studies with a control group and a modified version for studies without a control group (Slim et al., 2003).

We followed the guidelines of each assessment tool to determine the overall risk of bias for each study. Quality ratings for studies assessed with MINORS were assigned based on their scores. Studies with a control group were classified as high quality if they scored 19 or higher, fair quality if they scored between 13 and 18, and poor quality if they scored below 13. For studies without a control group, high quality was defined as a score of 13 or higher, fair quality ranged from 9 to 12, and poor quality was assigned to studies scoring below 8.

## Results

### Study characteristics

A total of 1,367 publications were identified through the initial search (Supplementary Figure S1). After duplicate removal and title/abstract screening, 54 articles underwent full-text review, of which 33 met the inclusion criteria. Among these, 16 were RCTs, including 4 crossover trials (1 pilot study) and 11 parallel trials (5 pilot studies). Fourteen studies were NRTs, with 2 utilizing a two-arm design and 12 employing a single-arm approach, including 1 pilot study. 1 NRT reported both long- and short-term outcomes in 2 separate papers. Additionally, 3 studies followed a retrospective design (Supplementary Table S2).

Sample sizes in the included studies ranged from 8 to 296 participants. The gender of ASD patients was not reported in 3 studies (Raghavan et al., 2022; Chen et al., 2024; Liu et al., 2023). Only 2 studies did not include female participants in their sample (Schmitt et al., 2023; Liu et al., 2019). At least one co-morbidity was present in ASD patients, with GI symptoms being the most frequently documented.

Eight studies reported patient compliance (Raghavan et al., 2022; Swanson et al., 2020; Sterne et al., 2019; Kong et al., 2021; Liu et al., 2023; Guidetti et al., 2022; Li et al., 2024; Niu et al., 2019); with 5 studies reporting a compliance rate greater than 90 percent (Sanctuary et al., 2019; Schmitt et al., 2023; Arnold et al., 2019; Palmer et al., 2025; Santocchi et al., 2020), and 1 study excluded patients with poor compliance (Li et al., 2024).

Fifteen studies examined the effect of probiotics, with 5 studies investigating the effect of a single probiotic strain (Mensi et al., 2021; Kong et al., 2021; Lin et al., 2024; Liu et al., 2019; Liu et al., 2023), while 10 studies investigated a combination of probiotic strains (Arnold et al., 2019; Shaaban et al., 2018; Santocchi et al., 2020; Guidetti et al., 2022; Li et al., 2024; Mazzone et al., 2024; Meguid et al., 2022; Niu et al., 2019; Sichel, 2013; Rojo-Marticella et al., 2025). Mainly, these interventions were *Bifidobacterium*, *Lactobacillus*, *Lactiplantibacillus,* and *Lacticaseibacillus* based; the most examined strains belonged to the species *Bifidobacterium longum*, *Lactobacillus acidophilus* (*n* = 5), and *Lactiplantibacillus plantarum* (*n* = 8). Additional probiotics examined included strains from the species *Lacticaseibacillus rhamnosus, Lacticaseibacillus casei, Lacticaseibacillus paracasei, Lactobacillus delbrueckii, Bifidobacterium longum, Bifidobacterium breve, Bifidobacterium animalis, Bifidobacterium bifidum, Streptococcus thermophilus*, and the strain *Bacteroides fragilis* BF839. The probiotics were administered in capsule or powder form with doses ranging from 0.5 billion to 900 billion colony-forming units (CFU).

Fewer studies examined prebiotics (*n* = 4), synbiotics (*n* = 5), and FMT (*n* = 9); additional information is provided in Supplementary Table S2. The treatment period ranged from 3 weeks to 15 months, and only 6 studies reported post-intervention follow-up results (Grimaldi et al., 2018; Wang et al., 2024; Li et al., 2024; Kang et al., 2019; Li et al., 2021; Li et al., 2024). A matching placebo or no treatment was used to compare the interventions.

Numerous scales are available for retrieving information about the presence or intensity of ASD-related behavioral and GI symptoms, which parents/caregivers or trained clinicians primarily answered. Multiple articles used more than one scale to assess ASD-related behaviors. The most commonly used assessments were ABC (*n* = 12), AuBC (*n* = 6), CARS (*n* = 10), SRS, including SRS-2 and SRS-Taiwan version (*n* = 14), and ATEC (*n* = 5). The AuBC is a tool consisting of 57 questions grouped into 5 subscales: Sensory, Relating, Body and Object Use, Language, and Social and Self-Help skills to be answered by a parent/caregiver, as the score increases autism severity increases (Krug et al., 1980). The ABC is a standardized tool consisting of 58 items across five subscales: Irritability, Lethargy/Social Withdrawal, Stereotypic Behavior, Hyperactivity/Noncompliance, and Inappropriate Speech. It is completed by parents, caregivers, or trained observers to assess maladaptive behaviors in individuals with developmental disabilities, including autism. Higher scores indicate greater severity of problem behaviors (Aman et al., 1985). Additionally, the CARS, consisting of 15 items, was the second most used standardized tool for children as young as 2 years of age, it is intended to be a direct observational tool conducted by trained professionals. Higher scores also indicate greater severity (Schopler et al., 1980).

As for GI symptoms, the most used tool was the Gastrointestinal Symptom Rating Scale (GSRS), consisting of 15 items assessing GI symptoms like abdominal pain, nausea and vomiting, heartburn, and many others. Each item is rated on a 7-point Likert scale, anchored at 0 (absence of symptoms) to 3 (extreme severity), with half-steps included to increase sensitivity (Svedlund et al., 1988). Many other tools were used like the Gastrointestinal severity index (GSI) (*n* = 2) and a modified version of it, the 6-GSI (*n* = 5), GI History survey (GIH) (*n* = 1), Bristol stool form scale (BSFS) (*n* = 4), PedsQL GI Module Scales (*n* = 1), Questionnaire on pediatric gastrointestinal symptoms– Rome III (QPGS-RIII) (*n* = 1). Also, 5 studies reported data with either unspecified questionnaires or qualitative GI diaries.

### Quality assessment

Most RCTs showed some concern when assessed for quality in terms of reporting the method of randomization, deviations from the intervention, missing outcome data, method of measurement of the outcome, and their selection of the reported results (Supplementary Figure S2). All studies used an intention-to-treat analysis (ITT) except for 3 which opted for a per-protocol analysis (PPS) (Liu et al., 2023; Lin et al., 2024; Liu et al., 2019). The method used for the random allocation of participants was reported in 11 out of the 14 RCTs (Grimaldi et al., 2018; Sanctuary et al., 2019; Liu et al., 2023; Schmitt et al., 2023; Palmer et al., 2025; Wang et al., 2024; Santocchi et al., 2020; Kong et al., 2021; Lin et al., 2024; Liu et al., 2019; Rojo-Marticella et al., 2025) and the remaining 3 articles did not specify their methods. A limitation in 3 of the RCTs was their small sample size with an average of 11 participants (Sanctuary et al., 2019; Schmitt et al., 2023; Arnold et al., 2019), these studies failed to identify any significant differences in the outcomes between the 2 treatment groups, possibly due to their lack of power. Only 5 RCTs scored a low risk of bias in all domains (Schmitt et al., 2023; Palmer et al., 2025; Santocchi et al., 2020; Mazzone et al., 2024; Rojo-Marticella et al., 2025).

The NRTs displayed suboptimal quality overall, with only 3 being classified as high quality (Li et al., 2024; Meguid et al., 2022; Niu et al., 2019). A limitation of most of these studies was the lack of a comparator for the analysis as only 1 study was two-armed (Niu et al., 2019). All NRTs were open-label, which introduces potential bias when assessing outcomes. Supplementary Table S3 summarizes the quality assessment of the NRTs. The rest of the studies employed a retrospective observational design, and their quality assessment is summarized in Supplementary Table S4. In addition to the lack of blinding, none of the observational studies included a true control group in their study design; therefore, their analyses were limited to intra-group comparisons.

### Effect of probiotics, prebiotics, and synbiotics on ASD-related behavioral outcomes

#### Probiotics

Fourteen studies assessed the effect of probiotics on outcomes in children with ASD, and 1 assessed the effect of probiotics on both the pediatric and adult populations (Kong et al., 2021). Of these, 9 were RCTs, all comparing intervention and placebo groups. More than half reported significant improvements in ASD-related behavioral outcomes, while 4 found no significant differences (Arnold et al., 2019; Santocchi et al., 2020; Liu et al., 2019; Rojo-Marticella et al., 2025).

Lin et al. conducted a parallel RCT in which they examined the role of *B. fragilis* BF839 strain on 60 children aged 2–10 years and found a significant decrease (improvement) in the body and object use subscale of the AuBC score at the end of the intervention period, which was more pronounced in children aged less than 4 years. Of note, in children with a baseline CARS score of more than 30 (cutoff score used to diagnose autism), they found a decrease in all of AuBC total score, its subscale body and object use, and CARS score (improvements). They also inferred that improving the GI symptoms of children with ASD can diminish autistic symptoms after finding a positive correlation between the AuBC score and the GSRS score (Lin et al., 2024).

Similarly, Liu et al. conducted a parallel RCT on 82 children aged 7–15, in which they divided the 2 intervention groups into early (E) and late (L). The E group was given the probiotic *Lp. plantarum* sp. for the whole duration of 4 months, while the L group was given a placebo for the first 2 months, followed by *Lp. plantarum* sp. in the following 2 months. They found a significant improvement in the ASEBA anxious/depressed score when comparing the 2 groups at the end of the first 2 months. The L group also showed a significant improvement in this subscale at the end of the 4 months. The E group did not sustain the improvement in this score however between the 2- and 4-month period, which is believed to be attributed to the influence of an outlier (Liu et al., 2023).

Mazzone et al. reported a significant improvement in the social behavior of ASD children in a parallel RCT investigating the effects of BioGaia Gastrus (*Limosilactobacillus reuteri,* DSM 17938 and ATCC PTA 6475 strains) on 43 children aged 5.8 (±1.3) years. Specifically, they found improvements in their total SRS score and its subscale of social communication. Additionally, and consistently with the SRS score improvement, they found an improvement in the social functioning subdomain of the ABAS-2 score (measured by the social adaptive composite score). This study also benefited from a 0% dropout rate from either group. Although the 3 previous studies showed promising results, they all suffered from a lack of female participant representation, and therefore a lack of a more comprehensive analysis in terms of gender-related variations (Mazzone et al., 2024).

In a different study design, Guidetti et al. conducted a crossover RCT on 61 children aged between 24 months and 16 years old in which they received either a probiotic mixture or a placebo for 3 months, followed by a 2-month washout period, and finally another 3-month opposite treatment period. They noticed improvements in both communication and disadaptive behavior through an increase in the VABS and PEP3 scores, respectively, as well as a decrease in the PSI scores. The study originally included 94 participants, of whom 25 have dropped out, and only 61 completed the follow-up. The high number of dropouts is believed to be attributed to the lack of a water-soluble form of the probiotic/placebo for children who have an issue with food selectivity (Guidetti et al., 2022).

Kong et al. investigated subjects aged 3–25 years old for the effect of *Lp. plantarum* PS128 strain in their parallel RCT in which they assigned a probiotic and a placebo group to receive their intervention for 28 weeks, in addition to oxytocin starting on week 16 for both groups. They found significant improvements in the CGI-I scores only for the probiotic + oxytocin group when compared to the control group. This study also found trends of improvements only in the combination treatment group in ABC and SRS scores, but none of them reached statistical significance (Kong et al., 2021).

On the other hand, Marticella et al. investigated the effects of probiotics on ASD symptoms in children aged 5–16 years using the SRS-2 and found no significant benefits in inter-group or intra-group analyses. Instead, the placebo group demonstrated a trend toward improvement. However, the study’s small sample size limited its statistical power, potentially affecting the reliability of the findings (Rojo-Marticella et al., 2025).

Of the 3 remaining RCTs, Arnold et al. and Santocchi et al. used the De Simone Formulation (*L. acidophilus* sp., *Lp. plantarum* sp., *Lc. casei* sp., *L. delbrueckii* subsp. *bulgaricus, B. longum* sp., *B. breve* sp., *B. infantis* sp., *S. thermophilus* sp.), and Liu et al. used *Lp. plantarum* PS128 strain as their probiotic of choice (Arnold et al., 2019; Santocchi et al., 2020; Liu et al., 2019). Arnold et al. performed a crossover RCT on a small sample of 10 children aged 3–12 years and found no significant outcomes, though his study mainly assessed the safety of this probiotic rather than its effect on behavioral outcomes (Arnold et al., 2019). Similarly, Santocchi et al. found no significant changes in overall behavioral outcomes, however in their secondary analysis, they found an improvement in the total ADOS-CSS score and its subset Social-Affect over the duration of 6 months in children with no GI symptoms. Additionally, the group that did have symptoms not only experienced an improvement in some of their GI symptoms (reported by Total GSI, Total 6-GSI, stool smell, and flatulence mean scores), they also found a positive effect on their adaptive functioning (Receptive Skills, Domestic Skills and Coping Skills VABS-II subscales) and sensory profiles (Multisensory Processing subscale) (Santocchi et al., 2020).

Liu et al. found no overall difference in the behavioral outcomes between the 2 groups. However, when measuring the differences within the probiotics group, they found that children aged 7–12 years experienced improvements in rule-breaking behavior, inattention, hyperactivity/impulsivity, and opposition/defiance, while those aged 13–15 did not (Liu et al., 2019).

In contrast to the RCTs, all 5 NRTs reported significant improvements in behavioral outcomes after the administration of probiotics (Shaaban et al., 2018; Li et al., 2024; Meguid et al., 2022; Niu et al., 2019; Sichel, 2013), and all interventions used were different combinations of *Lactobacillus* and *Bifidobacterium* probiotics.

In their open-label study conducted on participants aged 3–16 years, West et al. found that a 3-week course of probiotics decreased the overall ATEC scores along with its 4 subdomains (improvement). This study suffered from not having any set criteria for selection; instead, the participants volunteered themselves, possibly introducing selection bias. The design of this study was of poor quality; therefore, the results might not coincide with the average effect of treatment on the larger target population (Sichel, 2013). Shaaban et al. also reported a significant decrease in total ATEC scores in addition to all 4 of its subdomains for children aged 5–9 years (improvement) (Shaaban et al., 2018). Both studies suffered from their small sample sizes. A significant improvement in the total CARS score and several of its subdomains was seen in the study by Meguid et al. conducted on children aged 2–5 years, which lasted 3 months (Meguid et al., 2022). They also reported an improvement in anxiety after the 3 months; however, no *p*-values were reported for it. Similarly, Li et al. observed a significant reduction in CARS score by 15% following the intervention on children aged 3–12 years (Li et al., 2024).

Unlike the 4 previous studies, Niu et al. performed a two-arm trial on children aged 3–8 years comparing probiotics with behavioral training against behavioral training alone, with no probiotic-only group, rendering the beneficial effect of probiotics alone unexplored. They found a significant decrease in the total ATEC score and all 4 of its subdomains in the probiotic + behavioral training group (improvement). Although they mentioned the control group showed no significant changes in their ATEC scores, they did not provide any p-values for intergroup comparison of improvement. Overall, the clinical impact of these NRTs is limited due to the lack of blinding and randomization, their comparison to baseline variables only, and potential selection bias (Niu et al., 2019).

The last probiotic study applied a retrospective observational design in their real-world experiment conducted on participants aged 7.2 (± 3.43) months, in which they evaluated the changes in CGI score before and after taking *Lp. plantarum* PS128 strain (n = 105) or other probiotics (n = 26). They reported a significant improvement in the CGI-S scores of the PS128 group compared to the other probiotics; they also reported an improvement in their CGI-I scores, however, the p-value was not reported for it. The positive effects were more evident in younger children. It is important to note that due to the large difference in number between the 2 probiotic groups, comparing them fairly may be challenging due to statistical power imbalances, which could introduce bias (Mensi et al., 2021).

#### Prebiotics

Fewer studies have investigated the effect of prebiotics on behavioral outcomes in children with ASD, with 3 RCTs and 1 NRT available. Grimaldi et al. conducted a randomized four-arm trial on children aged from 4 to 11 years that lasted 6 weeks to investigate the effects of prebiotic supplementation containing GOS in children following exclusion diets (gluten- and casein-free) compared to those on unrestricted diets. In this study, the effect of the prebiotic cannot be fully isolated, as participants were also following a gluten- and casein-free diet. While the overall severity of behavioral traits remained unchanged, children receiving both the exclusion diet and prebiotic therapy exhibited reduced anti-social behavior (reported by reduced ATEC anti-social scale) along with improved social skills (reported by AQ questionnaire social-skills scale). Although this study started with 41 participants, only 26 participants completed the full intervention, reducing its statistical power to 80% (Grimaldi et al., 2018).

Using a similar intervention, Palmer et al. administered GOS to children aged 4–10 years for 6 weeks in their parallel RCT and did not find any significant improvements in behavioral scores (Palmer et al., 2025). In the RCT performed by Raghavan et al. on 18 participants, only 13 participants were included in their PPS, and all 18 were included in the ITT. In both analyses, the control groups did not achieve their endpoint improvement in CARS score of 4.5 points. In the intervention group receiving Nichi Glucan (black yeast-derived AFO-202 beta-glucan), 25% achieved the endpoint in ITT, and 33% achieved it in PPS. Although neither group’s proportion reached 50%, they found a significantly higher proportion of the CARS score improvement endpoint in the Nichi Glucan group. This study suffered from many design flaws, namely the limited number of participants, unequal distribution of genders, and their distribution imbalance in the number of participants between groups. This study was also open-label, with only the assessor being blinded to the intervention, possibly introducing biased results (Raghavan et al., 2022).

Finally, an exploratory NRT, open-label, single-arm trial on children aged 4–9 years investigated the effect of partially hydrolyzed guar gum and reported a decrease in the irritability subscale of the ABC score of children compared to their baseline (improvement). However, they also suffered from a small sample size of 13, in addition to the lack of a control group (Inoue et al., 2019).

#### Synbiotics

Sanctuary et al. conducted a double-blind crossover RCT on children aged 2–11 years in which they investigated the effects of combination therapy [bovine colostrum product (BCP) as a source of prebiotic oligosaccharides combined with *B. longum* subsp. *infantum* (UCD272)] against prebiotics alone (BCP only). In the BCP-only group, a significant reduction in ABC irritability, lethargy, stereotypy, hyperactivity, and total score (improvement) was noticed when compared to their baseline values, while in the combination therapy group, they noticed only a decrease in their ABC-lethargy scores. When the 2 groups were compared against one another, only the ABC-stereotypy score was seen to be significantly improved in the BCP-only group. No other significant results were reported. This study suffered from several limitations. First, their sample size was small (*n* = 8), limiting the generalizability of the findings. Additionally, the study lacked a clear control group receiving placebo, or a group receiving probiotics only. Moreover, over half of their sample had irritable bowel syndrome, which further complicates the interpretation of their results (Sanctuary et al., 2019).

Another double-blind RCT investigating the effects of FOS combined with a probiotic mixture on children aged 3–9 years found significant improvements in overall symptom severity, as measured by the total ATEC score. The study also reported reductions in impairment severity within the domains of speech, language, communication, and sociability after 60 to 108 days of treatment (Wang et al., 2020). Although the trial included a placebo-controlled design, it only analyzed within-group differences between baseline and post-intervention measures, without conducting direct comparisons between the treatment and placebo groups.

In a recently published phase Ib, double-blind, crossover RCT, Schmitt et al. investigated the effect of SB-121 (a combination of *Li. reuteri* sp., Sephadex® (dextran microparticles; 200 mg), and maltose). The participants received 28 days of probiotic/placebo, followed by 14 days of washout, then another 28 days of the opposite treatment. Participants receiving SB-121 showed significant improvements in adaptive behavior, as measured by the VABS-3 score, and a trend toward enhanced social preference as measured by eye tracking, when compared to baseline. The study, however, found no significant improvements in behavioral scores in intergroup comparisons. This is one of the few studies that included participants aged 15–27 years in its design. Although this study design had a low risk of bias, it suffered from a lack of female representation and a small number of participants (*n* = 15) (Schmitt et al., 2023).

Phan et al. conducted an open-label study of 296 participants aged 10.41 (±7.14) years, 170 of whom completed a 3-month follow-up after receiving their synbiotics. The study reported no significant changes in any of the behavioral tools. Although parental Global Impressions (PGIA) scores suggested perceived benefits for roughly half of the participants, these findings, obtained without a placebo control, raise the possibility of placebo effects. This study suffered from a high drop-out rate, which biases results toward participants who perceive greater improvement (Phan et al., 2024).

Similarly, Mitchell et al. conducted a 12-week open-label study on children aged between 5 and 11 years to assess the impact of synbiotics, with and without gut-directed hypnotherapy (GDH), in autistic children with comorbid disorders of gut-brain interaction (DGBI). Autism-related symptoms were assessed using ABC and the Parental Reported Autism Symptoms Scale for Autism Spectrum Disorder (PRAS-ASD). The synbiotic-only group demonstrated significant reductions in two ABC subscales from baseline: Stereotypic Behavior and Hyperactivity/Non-compliance. While the study did not find a significant reduction in anxiety scores for this group, it did note a trend toward significance for reductions in irritability scores. These results indicate that synbiotic supplementation alone can have a beneficial effect on certain behavioral symptoms in this population (Mitchell et al., 2025).

### Effect of probiotics, prebiotics, and synbiotics on GI outcomes

Most studies assessed GI health both before and after treatment. Five probiotic studies reported no changes in gut health (Arnold et al., 2019; Santocchi et al., 2020; Kong et al., 2021; Mazzone et al., 2024; Sichel, 2013), and although Lin et al. did not find any significant overall differences in GI health, the subgroup analysis of children with a baseline CARS score of more than 30 showed significant improvements in their GSRS score. They also observed a significant positive correlation between the CARS score and GSRS score, indicating that greater autism severity was associated with more severe GI symptoms (Lin et al., 2024).

Guidetti et al. (2022), Niu et al. (2019), Li et al. (2024), and Shaaban et al. (2018) all found significant improvements in gut health, with Shaaban et al. reporting a strong correlation between improvements in autism severity and GI symptom severity (Shaaban et al., 2018), similar to Lin et al. (2024). Niu et al. (2019) mentioned that the behavioral improvement in children with GI problems was more noticeable compared to children with no GI problems, but there was no mention of the statistical significance of this observation. Meguid et al. (2022) also mentioned relief from several abdominal discomforts and stool consistency improvements, with no mention of their statistical significance.

Of the 4 prebiotic studies, 3 investigated GI health (Inoue et al., 2019; Grimaldi et al., 2018; Palmer et al., 2025). Inoue et al. found that supplementation of partially hydrolyzed guar gum significantly increased defecation frequency in all children who had constipation (Inoue et al., 2019). Neither Grimaldi et al. (2018) nor Palmer et al. (2025) found significant changes in GI outcomes; however, Grimaldi et al. (2018) found a non-significant general trend toward improvement in symptoms after prebiotic use, and Palmer et al. noticed a reduction in the proportion of children with severe GI symptoms following GOS therapy (Palmer et al., 2025).

Phan et al., Wang et al. and Mitchell et al. all found significant improvements in GI symptom severity following synbiotic supplementation (Wang et al., 2020; Phan et al., 2024; Mitchell et al., 2025). Wang et al. (2020) observed reductions in constipation, diarrhea, and stool smell subscales, as well as a decrease in the total 6-GSI score. Phan et al. (2024) reported a significant reduction in participants’ GSRS scores. Mitchell et al. (2025) also found significant improvements in GI symptoms among autistic children with comorbid disorders of gut-brain interaction (DGBI) after synbiotic supplementation. Sanctuary et al. (2019), while not observing significant intergroup differences, found that 7/8 participants in the BCP-only group and all participants in the combination therapy group experienced improvements in their GI symptoms.

### Effect of FMT on ASD-related behavioral outcomes

There were 1 RCT, 6 NRTs (all open-label clinical trials), and 2 retrospective studies investigating the effect of FMT therapy on ASD, and they all showed significant improvements in the overall severity of ASD (Chen et al., 2024; Liu et al., 2023; Wang et al., 2024; Li et al., 2024; Kang et al., 2019; Li et al., 2021; Li et al., 2024; Pan et al., 2022; Zhang et al., 2022; Kang et al., 2017).

Wang et al. conducted an RCT involving 41 children with ASD to compare the effects of FMT with a placebo. Participants receiving FMT showed notable reductions in symptoms as measured by CARS, SRS, and ABC. However, several factors could limit the results. The small sample size may reduce the generalizability of the findings (Wang et al., 2024). Additionally, the study’s duration might have been too short to observe long-term effects. Variations in individual microbiota compositions and responses to FMT could also affect the consistency of the results. Finally, the placebo effect and potential biases in reporting and assessment could influence the observed outcomes.

Another clinical trial, reported in 2 papers (Kang et al., 2019; Kang et al., 2017) examined FMT in 18 individuals with ASD and GI symptoms. Treatment began with a 2-week vancomycin regimen and bowel cleanse, followed by high-dose FMT via oral or rectal administration. A lower maintenance dose continued for 7–8 weeks. After the 10-week treatment, participants were monitored for 8 weeks. Compared to baseline, ASD-related symptoms (PGI-III, CARS) and overall severity improved significantly, with effects maintained at follow-up. SRS and ABC assessments also showed reductions in social deficits, irritability, hyperactivity, lethargy, stereotypy, and aberrant speech. Two years after treatment, PGI-III, CARS, SRS, and ABC scores indicated continued improvement.

Furthermore, Li et al. recruited 40 children. The study duration was 12 weeks, which consisted of a 4-week FMT therapy phase and a follow-up observation phase for 8 weeks after treatment. Subjects received 2 liters of polyethylene glycol the night before FMT and remained fasting until the scheduled treatment. FMT led to significant improvements in ASD symptoms. ABC scores significantly decreased post-treatment, with continued improvement observed up to 8 weeks after FMT. CARS scores showed a 10% decrease (improvement) at the end of treatment, maintaining a 6% decrease in the 8-week follow-up. The SRS also showed improvements immediately after treatment, yet these benefits diminished after 8 to 12 weeks. Additionally, the SAS indicated that parents’ anxiety levels improved in parallel with reductions in GI and autism-related symptoms, but these effects returned to baseline by the 8-week mark. Findings suggest that sustained or repeated treatment may be necessary to maintain long-term benefits (Li et al., 2021).

Another study by Chen et al. administered ASD patients freeze-dried microbiota capsules equivalent to 200 g fresh stool for 4 months, each month there was a session that was initiated by clinical assessments then 12-day oral administration of the capsules. Results showed improvements in cognitive deficiency, as AuBC and CARS scores significantly decreased compared to baseline after 3 months of treatment. It was also observed that for younger age groups (2–4 years) better responses in the AuBC and CARS were seen at 2 and 3 months post-FMT (Chen et al., 2024). A study by Liu et al. conducted on children younger than 2 years of age showed that ABC scores showed a persistent reduction after washed microbiota transplantation (WMT), indicating symptom amelioration. The reductions in ABC scores across the four WMT treatments were statistically significant. Pearson correlation analysis revealed a significant positive correlation between ABC scores and sleep disturbance, suggesting a strong link between behavioral and sleep quality improvements. All outcomes were relative to the baseline (Liu et al., 2023).

Li et al. conducted a study on 98 children and observed significant improvements in core ASD symptoms among children who received FMT. Participants were assessed using the AuBC, CARS, and, SRS. The results showed that children who received FMT through oral capsules and nasal jejunal tubes had greater reductions (improvement) in their AuBC, CARS, and SRS scores compared to those who received FMT through the transendoscopic enteral tube (TET) method. These improvements were evident both immediately after treatment and at the 8-week follow-up (Li et al., 2024).

Li et al. conducted a study on 38 children who were administered oral lyophilized FMT treatment (a ratio of 1 g donor stool per 1 kg of recipient body weight) once every 4 weeks for a total of 12 weeks and were followed up at week 20. AuBC, CARS, SRS, and SDSC scores showed a significant 20, 10, 6, and 10% decrease (on average) relative to baseline (improvement), respectively. All these studies had a small sample size, which may affect the generalizability of the studies on different populations (Li et al., 2024).

In the retrospective study by Pan et al., 42 children were recruited to receive 2–5 courses of WMT with treatment duration varying according to the number of courses completed and assessments were conducted at baseline and after each treatment. ABC and CARS scores were reduced significantly after WMT (improvement), and enhanced improvements were seen after additional courses. Between reductions in ABC and SDSC scores a significant positive correlation was seen, indicating a connection between improved sleep and behavioral symptoms (Pan et al., 2022).

Another retrospective study by Zhang et al. included 49 children and investigated WMT effects on behavioral symptoms. The study divided participants into constipation (*n* = 24) and no constipation (*n* = 25) groups (control group). For the constipation group, the change in the CARS scale of W1 was not significant, yet W2 was statistically significant relative to the baseline (improvement). For the AuBC scale, the constipation group was statistically distinguished from the baseline after W2 (improvement); however, for the control group, no significance was found after W1 and W2 (Zhang et al., 2022).

### Effect of FMT on GI outcomes

Wang et al. observed significant improvements in GI symptoms following FMT intervention. Participants in the FMT group experienced notable reductions in GI symptoms, particularly in areas such as diarrhea and constipation. The average GSRS scores decreased significantly, indicating a marked improvement in overall GI health (Wang et al., 2024).

Regarding Kang et al. patients suffering from chronic constipation and/or diarrhea when recruited showed more frequent bowel movements post-FMT therapy. At the end of the intervention and the 2-year follow-up, relative to baseline, there was a 58% reduction in GSRS (on average), and for abnormal stools, a 26% reduction in percent days was noted (improvements). All sub-categories of GSRS and daily stool records observed improvements. A positive significant correlation in the percentage changes in GSRS scores was seen to be correlated with CARS, SRS, and ABC scores, however, daily stool records (a GI assessment tool) showed no significant correlation with behavioral severity (Kang et al., 2019; Kang et al., 2017). In the study by Li et al. FMT significantly improved GI symptoms in children, as measured by the GSRS. Following 4 weeks of FMT treatment, GSRS scores in participants were reduced by 35%, with the improvement lasting for at least 8 weeks post-treatment. This reduction indicates a significant alleviation of symptoms such as abdominal pain, reflux, indigestion, diarrhea, and constipation. Moreover, stool consistency improved, with a significant reduction in the defecation of hard stools (type 1 or 2) and soft/liquid stools (type 6 or 7) (Li et al., 2021).

Moreover, Chen et al. showed significant improvements in GSRS and BSFS compared to the baseline 3 months post-FMT. They also compared two age subgroups and found enhanced overall improvements in GSRS 2 and 3 months post-treatment. Liu et al. showed increased BSFS scores post-WMT, reflecting improved GI function and constipation relief, with significant *p*-values at each WMT relative to baseline (Chen et al., 2024).

Li et al. showed that both the oral capsule and nasal jejunal tube groups experienced notable reductions in GI symptoms such as abdominal pain, reflux, indigestion, diarrhea, and constipation. These improvements were observed both immediately after treatment and at the 8-week follow-up. The TET group also showed improvements but to a lesser extent compared to the other two methods (Li et al., 2024).

Furthermore, Li et al. studied 38 children, of which 31 had GI symptoms. Significant improvements were found in the average score of GSRS which decreased by 51% relative to baseline (Li et al., 2024). The study by Pan et al. had 50% of its participants exhibiting constipation at baseline, which was significantly decreased after WMT approaching zero following the fourth WMT. Post-WMT, the amount of children with normal stool consistency increased to 70.27% (baseline = 51.35%) (Pan et al., 2022). Zhang et al. reported that after WMT treatment, in the constipation group, a very significant difference in the BSFS of W1 and W2 was found (improvement) (Zhang et al., 2022).

### Adverse events

Major adverse events (AE) were not reported in any of the included studies. The main AEs reported were clinically minor, relating to GI abnormalities, most commonly diarrhea. It was reported in 6 probiotic studies (Shaaban et al., 2018; Mensi et al., 2021; Lin et al., 2024; Guidetti et al., 2022; Mazzone et al., 2024; Niu et al., 2019), 2 prebiotic studies (Raghavan et al., 2022; Grimaldi et al., 2018), and 2 synbiotic studies (Sanctuary et al., 2019; Schmitt et al., 2023). For FMT trials, it was generally safe, and minimal adverse effects were reported. Two trials reported hyperactivity and aggression (Li et al., 2021; Kang et al., 2017) which were temporary. Three studies used Washed microbiota transplant which significantly decreases AEs caused by FMT via removing contaminants through a series of automated washing procedures (Liu et al., 2023; Pan et al., 2022; Zhang et al., 2022).

## Discussion

This systematic review provides the most comprehensive analysis of probiotics, prebiotics, synbiotics, and FMT for individuals with ASD. Previous reviews have primarily focused on probiotics and were limited to randomized controlled trials, overlooking valuable data from other microbiome-modulating interventions and diverse study methodologies. By including a broader range of interventions and study designs, our review offers a more complete understanding of the potential role of microbiota-based therapies in ASD, providing new insights that can inform clinical practice and guide future research.

Our findings are consistent with previous systematic reviews, which reported that prebiotics may improve certain behavioral outcomes, such as reductions in anti-social behavior, while showing inconclusive evidence for GI benefits, with some studies noting improvements such as increased frequency of weekly defecation (Gao et al., 2025; Yang et al., 2020). Regarding synbiotics, prior studies have demonstrated improvements in GI symptoms, particularly reductions in the total 6-GSI score, as well as significant improvements in irritability and stereotypy in participants receiving BCP alone. These benefits may be attributable to anti-inflammatory effects, including reductions in interleukin-13 (IL-13) and tumor necrosis factor-alpha (TNF-*α*) levels (Azari et al., 2024; Lu et al., 2025).

Our findings also suggest that FMT exhibits the most consistent and long-lasting benefits, particularly in improving both behavioral and GI symptoms in ASD. Probiotics demonstrated moderate improvements in social behavior and adaptive functioning, but their efficacy varied across studies. Prebiotics and synbiotics showed mixed results, with some evidence of GI and behavioral improvements, but a lack of robust, reproducible effects. These findings indicate that while microbiota-targeted therapies hold promise, their efficacy remains dependent on specific formulations, treatment durations, and patient characteristics.

The inconsistent findings regarding behavioral and GI symptoms observed across studies evaluating probiotics, prebiotics, and synbiotics may have arisen from several factors. These included differences in the specific interventions used, variations in sample sizes, the age of participants, and the duration of treatment and follow-up. Such variability may have led to divergent outcomes, making it challenging to draw definitive conclusions. These factors highlighted the need for more standardized protocols in future studies to reduce variability and better assess the efficacy of microbiome-based therapies in individuals with ASD.

A previous meta-analysis of RCTs demonstrated that probiotics significantly improved overall behavioral symptoms in the probiotic group compared to the control group, which aligns with our findings (Lee et al., 2024). Their subgroup analysis of 5 RCTs further showed that only multiple-strain probiotics, rather than single-strain formulations, led to significant improvements in overall ASD symptoms, a trend that we also observed in the studies included in our review.

Furthermore, a systematic review by Dossaji et al. investigating the impact of FMT on both behavioral and GI symptoms reported significant improvements in both domains, supporting our findings. Unlike their review, which lacked RCTs assessing FMT, our analysis includes RCT evidence, strengthening the understanding of FMT’s potential benefits. Nevertheless, further well-designed RCTs with larger sample sizes remain essential to confirm their efficacy in children with ASD (Dossaji et al., 2023).

Our analysis highlights strain-specific effects of probiotic interventions in ASD, with *Lp. plantarum* PS128 and *Li. reuteri* sp. showing consistency in behavioral improvement, particularly in social responsiveness and anxiety reduction. These findings align with behavioral improvements in multiple mouse models of ASD, where *Li. reuteri* sp. showed improvement by inducing oxytocin-dependent behavioral improvement (Buffington et al., 2016; Sgritta et al., 2019; Varian et al., 2017). Similarly, *Lp. plantarum* PS128 strain has been shown to influence dopaminergic and serotonergic pathways in mouse models potentially ameliorating behavioral dysregulation in ASD (Lu et al., 2021). In contrast, *B. longum* sp. and *L. acidophilus* sp. were significantly associated with improvements in GI outcomes, including reductions in abdominal pain and stool inconsistency. Demonstrated a reduction in abdominal pain severity and symptomology with *L. acidophilus* sp., matching our findings (Martoni et al., 2020). The mechanism of improving GI symptoms remains unclear; however, Cao et al. hypothesized that it could be by upregulating serotonin transporter expression in intestinal cells (Cao et al., 2018).

Numerous studies demonstrated that probiotics enhance the cognitive abilities of the brain in various conditions, including Alzheimer’s and major depressive disorder (Rudzki et al., 2019; Tamtaji et al., 2019). It is well established that a balanced GIT microbiome significantly affects our overall health. Studies showed a correlation between dysregulated gut microbiota and several GI and neurodegenerative diseases (Cryan et al., 2019). Pre-clinical studies have further linked disruptions to the brain-gut-microbiome axis to neurological and psychiatric diseases, including ASD (Martin et al., 2018), all of which highlight the importance of the bidirectional communication of the gut-brain axis.

An increase in the *Bacillota*/*Bacteroidota* ratio due to a decrease in *Bacteroidota* abundance was found to be significantly linked to ASD (Strati et al., 2017), which suggests that a dysbiotic state could contribute to the pathogenesis of autism, possibly by increasing metabolites that ultimately impact the brain and its development (De Angelis et al., 2015; Fowlie et al., 2018; Siniscalco et al., 2018). Therefore, altering the gut microbiota through the use of probiotics, prebiotics, synbiotics, or FMT could serve as a potential treatment for ASD.

Recent studies have begun to investigate whether improvements in autism symptoms are associated with specific microbial changes. A pediatric trial of lyophilized FMT reported that reductions in CARS, SRS, and ABC scores were associated with increased relative abundance of *Fusicatenibacter* and *Erysipelotrichaceae_*UCG-003, as well as shifts in fungal taxa (Li et al., 2024). Taken together, these data suggest that while symptom improvement can occur independent of obvious compositional shifts, specific bacterial and fungal taxa may modulate or reflect behavioral outcomes in some children with ASD.

With the well-known importance of gut barrier integrity for preventing systemic inflammation, and the association found between dysbiosis and abnormal neurotransmitter signaling in the brain of a mouse model of ASD (Resta, 2009), the anti-inflammatory abilities of some probiotics (Alipour et al., 2014; Kullisaar et al., 2003) come into play in a way that might be beneficial in maintaining neurological functions via maintaining the integrity and permeability of the gut barrier and reestablishing eubiosis (Lee et al., 2024; Hsiao et al., 2013).

Another important consideration is that the vagus nerve exerts many effects on the GIT that could alter the microbiome. Thus, targeting the vagal tone via modulating the microbiota could reestablish homeostasis to the microbiome-gut-brain axis (Bonaz et al., 2018). Moreover, some probiotic formulas were found to increase the production of some neurotransmitters such as GABA, dopamine, and serotonin, which can exert various effects either via epigenetic modifications or by acting as a ligand to their respective receptors (Qin and Wade, 2018).

Taken together, the improvements observed across behavioral and GI scales warrant careful interpretation. In our analysis, improvements in behavioral domains sometimes occurred alongside GI benefits, supporting the hypothesis of a bidirectional gut–brain axis whereby alleviating GI dysfunction may secondarily improve behavioral outcomes. However, strain-specific effects (e.g., *Li. reuteri* sp. through oxytocin modulation or *Lp. plantarum* PS128 strain via dopaminergic/serotonergic pathways) suggest that probiotics may also exert direct effects on the central nervous system, independent of GI changes. Prior studies showing behavioral improvement without consistent GI benefits support this possibility. Thus, it is likely that both direct neuroactive effects of specific strains and indirect effects mediated through gut health contribute to the observed variability. This dual mechanism may explain why different studies report divergent results and underscores the need for standardized protocols and mechanistic investigations in future trials.

### Future directions

Future research should thoroughly evaluate the variations that exist between patients within the same cohort, as well as between study cohorts, as ASD manifests with a wide spectrum of disease presentations and levels of disability, contributing to its heterogeneous nature. Ben Itzchak & Zachor, described that treatment outcomes could be influenced by patients’ demographics and characteristics (Itzchak E and Zachor, 2011). Furthermore, the male predominance in the included studies may fail to account for gender differences in the treatment response and presentation, as animal research suggests that the microbiota modulatory effects on the central nervous system (CNS) are gender-specific (Clarke et al., 2013). Notably, none of the studies included accounted for the use of psychotropic medications and the potential effects of classical drug treatments of patients with ASD, despite their roles as potential influencers on treatment outcomes and microbial composition. Additionally, comprehensive dietary data were limited, making it difficult to assess potential confounders such as the impact of nondigestible carbohydrate intake on microbiota and ASD-related symptoms.

Given the optimistic potential of prebiotics and synbiotics, greater efforts are encouraged to assess their effectiveness, as these interventions could have a broader effect on microbial diversity, greater safety, and wider acceptability across population groups. Identifying the most effective prebiotics for the growth of key bacterial species associated with ASD should be a central focus of upcoming studies. In addition, it is not well-established which product among prebiotics, probiotics, or synbiotics is better for symptoms of ASD. Therefore, more studies are required to explore various synbiotic combinations and dosages while comprehensively comparing their microbial composition and biological functions against prebiotics alone, probiotics alone, and control groups.

Liu et al., Lin et al., and Chen et al. found that younger children treated with PS128 experienced greater benefits than the older ones, *Ba. fragilis* BF839 behavior improvement was more pronounced in children aged less than 4 years, and freeze-dried microbiota capsules led to significant cognitive improvements, respectively (Chen et al., 2024; Lin et al., 2024; Liu et al., 2019). These findings are confirmed by previous studies highlighting that younger child’s age at the start of intervention is linked to better treatment outcomes (Itzchak E and Zachor, 2011; Vivanti et al., 2014). Early treatment with probiotic, prebiotic, synbiotic, or FMT interventions aimed at enhancing development in children susceptible to ASD could turn into a crucial research domain. Establishing a healthy gut during infancy to support healthy CNS development during critical periods may be more cost-effective and beneficial than long-term supplementation later in life, as the microbiota establishes early, and interventions at later stages may be both costly and require prolonged use.

### Strengths and limitations

Our study had several limitations that should be taken into consideration before interpreting the results. To begin with, the high heterogeneity in outcome measures, treatment regimens, formulations, dosages, lengths of treatment, and administration methodologies across studies makes the current evidence for the efficacy of probiotics, prebiotics, synbiotics, or FMT inconsistent. This variability limited our ability to conduct a meta-analysis, and thus deriving pooled effect estimates was not feasible. Moreover, the low methodological qualities of the published studies, small sample sizes, and lack of control groups restrict the conclusions that can be drawn from this review. Due to heterogeneity in outcome measures and incomplete reporting of subgroup-specific results across the included studies, we were unable to group findings according to intervention type, population characteristics, or demographics without introducing assumptions beyond the available data. Furthermore, insufficient age-stratified data prevented us from assessing how age influences the efficacy of microbiota-based interventions, underscoring the need for future research in this area.

Despite these limitations, this review has several strengths that should be addressed as well. To start with, our systematic approach involved a critical evaluation of the included studies using multiple validated assessment tools tailored to the appropriate study designs. Furthermore, the study search and data synthesis were conducted according to predefined criteria, using a piloted data extraction form reviewed by two independent reviewers. Another strength of this review is that the majority of studies included opted for an intention-to-treat analysis, maintaining an unbiased and more reliable assessment of treatment effectiveness.

## Conclusion

This systematic review highlights the potential of microbiome-targeted therapies for improving both behavioral and GI symptoms in individuals with ASD. FMT, in particular, consistently showed the most positive results across the studies included in our review, especially in reducing ASD severity and improving GI function. Probiotics showed moderate benefits in some studies, especially in enhancing social behaviors and adaptive functioning, but results were inconsistent. Prebiotics and synbiotics yielded mixed findings, with some evidence of behavioral and GI improvements but lacking robust, reproducible effects.

Moving forward, large-scale, well-controlled trials with standardized protocols, diverse populations, and long-term follow-ups are needed to establish definitive clinical guidelines. Additionally, personalized approaches based on microbiome profiling may enhance treatment efficacy by tailoring interventions to individual gut microbiota compositions. Overall, while microbiome-based interventions show promise in ASD management, further research is required to refine their therapeutic potential, optimize treatment strategies, and fully understand the mechanisms underlying their effects.

## Data Availability

The original contributions presented in the study are included in the article/Supplementary material, further inquiries can be directed to the corresponding author/s.
