# Supplementary material for: Microbiota-based interventions for autism spectrum disorder: a systematic review of efficacy and clinical potential
Source: Front Microbiol. 2025 Sep 26;16:1648118. doi: 10.3389/fmicb.2025.1648118 (PMC12512243; doi:10.3389/fmicb.2025.1648118)
Supplement: Supplementary file 1 [file Supplementary_file_1.docx]

**Appendix 1 – Supplementary Figures**

**Identification of studies via databases and registers**

Records removed *before screening*:

Duplicate records removed (n = 101)

Records marked as ineligible by automation tools (n = 0)

Records removed for other reasons (n = 0)

Records identified from:

PubMed (n = 1016)

Cochrane (n = 191)

Scopus (n = 160)

**Identification**

Records screened

(n = 1266)

Records excluded**

(n = 1212)

Reports sought for retrieval

(n = 54)

Reports not retrieved

(n = 0)

**Screening**

Reports excluded:

Wrong outcome (n = 7)

Wrong publication type

(n = 7)

Wrong study design (n = 2)

Wrong intervention (n = 2)

Wrong population (n = 2)

Not in English (n = 1)

Reports assessed for eligibility

(n = 54)

Studies included in review

(n = 33)

**Included**

**Figure.S1** Flow diagram of the screening process

|  | | **Randomization** | **Deviations from the intended interventions** | **Missing outcome data** | **Measurement of the outcome** | **Selection of the reported  result** | **Overall risk-of-bias** |
| --- | --- | --- | --- | --- | --- | --- | --- |
| **Palmer, 2024** | | 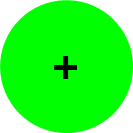 | 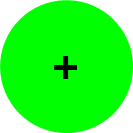 | 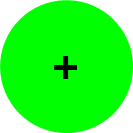 | 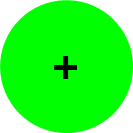 | 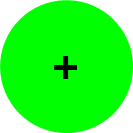 | 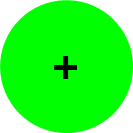 |
| **Lin, 2024** | | 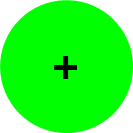 | 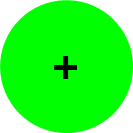 | 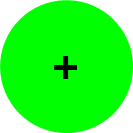 | 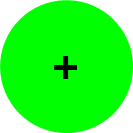 | 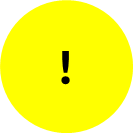 | 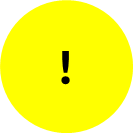 |
| **Grimaldi, 2018** | | 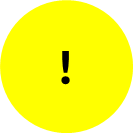 | 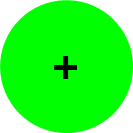 | 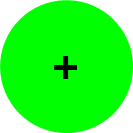 | 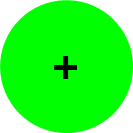 | 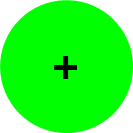 | 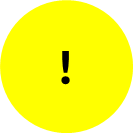 |
| **Raghavan, 2022** | | 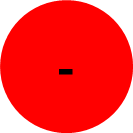 | 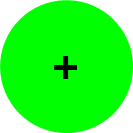 | 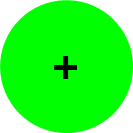 | 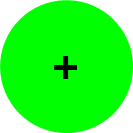 | 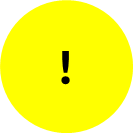 | 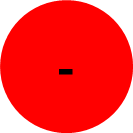 |
| **Guidetti, 2022** | | 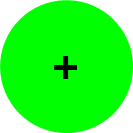 | 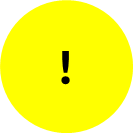 | 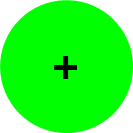 | 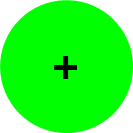 | 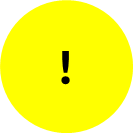 | 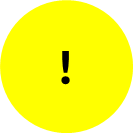 |
| **Rojo‑Marticella, 2025** | 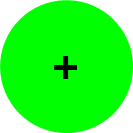 | 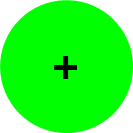 | 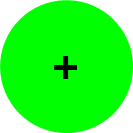 | 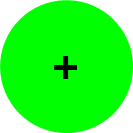 | 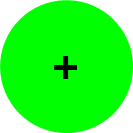 | 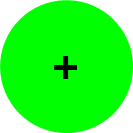 |  |
| **Kong, 2021** | | 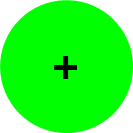 | 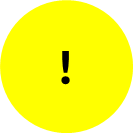 | 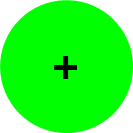 | 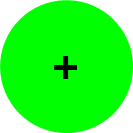 | 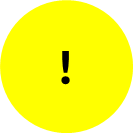 | 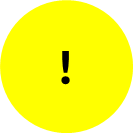 |
| **Wang, 2020** | | 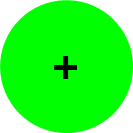 | 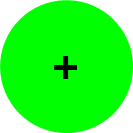 | 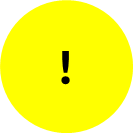 | 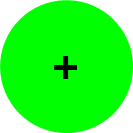 | 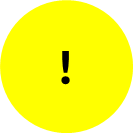 | 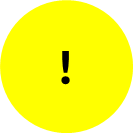 |
| **Wang, 2024** | | 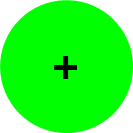 | 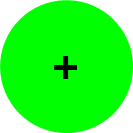 | 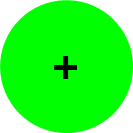 | 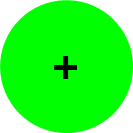 | 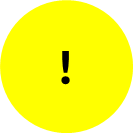 | 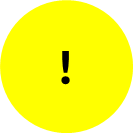 |
| **Mazzone, 2023** | | 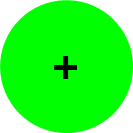 | 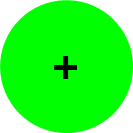 | 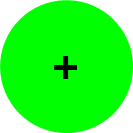 | 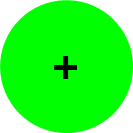 | 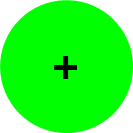 | 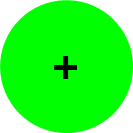 |
| **Sanctuary, 2019** | | 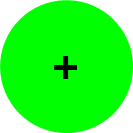 | 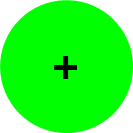 | 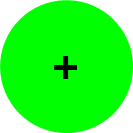 | 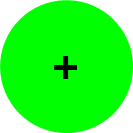 | 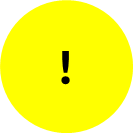 | 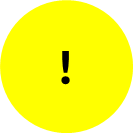 |
| **Santocchi, 2020** | | 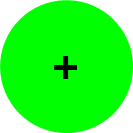 | 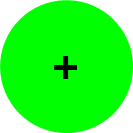 | 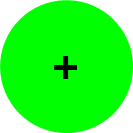 | 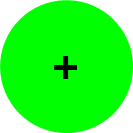 | 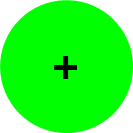 | 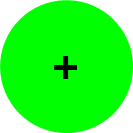 |
| **Schmitt, 2023** | | 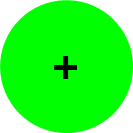 | 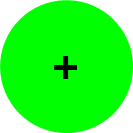 | 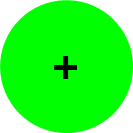 | 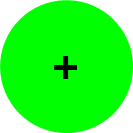 | 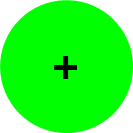 | 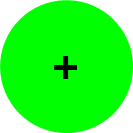 |
| **Arnold, 2019** | | 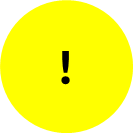 | 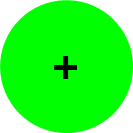 | 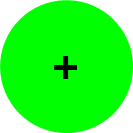 | 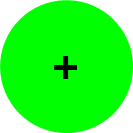 | 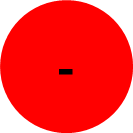 | 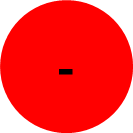 |
| **Liu, 2019** | | 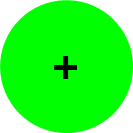 | 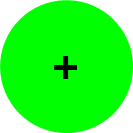 | 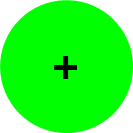 | 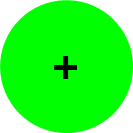 | 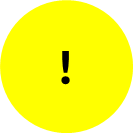 | 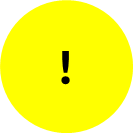 |
| **Y.-W**.**Liu, 2023** | | 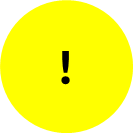 | 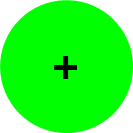 | 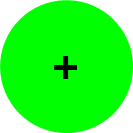 | 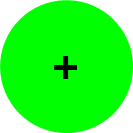 | 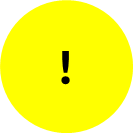 | 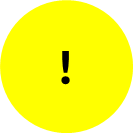 |

**Figure.S2** Results of the quality assessment of randomized controlled trials using the revised Cochrane risk-of-bias tool for randomized trials. + denotes “low risk”; ! denotes “some concerns”; and – denotes “high risk”. The trial was deemed to be at high risk of bias if at least one of its domains was rated as high risk. The trial was deemed to have a low risk of bias if all its domains were rated as low risk. Otherwise, the trial’s risk of bias is deemed questionable and denoted as having some concerns.

**Appendix 2 – Supplementary Tables**

**Table S1**: Search strategy across different databases

| Database Search Strategy | |
| --- | --- |
| Scopus | TITLE-ABS-KEY ( ( "developmental disorder" OR autism OR asd OR "pervasive developmental disorder" OR kanner OR ( language W/4 ( delay OR disability OR disorder ) ) OR ( ( communication OR speech ) W/4 disorder ) OR ( asperger AND NOT aspergillus ) ) AND ( microbiota OR microbiome OR "Gastrointestinal Microbiome" OR microbiotica OR metagenome OR ( ( gut OR gastrointestinal OR intestine ) AND ( flora OR microflora OR microbiota ) ) OR prebiotic OR probiotic OR synbiotic OR "Lactobacillus" OR lactobacteria OR "Bifidobacterium" OR bifidobacteria OR "Lactococcus" OR saccharomyces OR "Saccharomyces" OR streptococcus AND thermophilus OR "lactic acid bacteria" OR "bacillus subtilis" OR yoghurt OR yoghourt OR yogurt OR "Fecal Microbiota Transplantation" OR fmt OR ( ( feces OR faeces OR fecal OR stool OR microbiome OR microflora OR microbiota ) W/3 ( transplantation OR transfer OR enema OR donor OR transfusion ) ) OR "fecal bacteriotherapy" OR "faecal bacteriotherapy" ) ) AND NOT TITLE-ABS-KEY ( review OR "SYSTEMATIC REVIEW" OR "META-ANALYSIS" OR "CASE REPORT" OR letter OR comment ) |
| Cochrane Library | ("Child Development Disorders, Pervasive" OR "Schizophrenia, Childhood" OR "childhood schizophrenia" OR "Speech Disorders" OR autis* OR ASD OR "pervasive developmental disorder*" OR "kanner*" OR (language NEAR/4 (delay* OR disabilit* OR disorder* OR impair*)) OR ((communicat* OR speech) NEAR/4 disorder*) OR (asperg* NOT aspergill*)) AND (microbiota* OR microbiome* OR "Gastrointestinal Microbiome" OR microbiotica OR metagenome* OR ((gut OR gastrointestinal OR intestin*) AND (flora OR microflora OR microbiota)) OR "gut brain axis" OR prebiotic* OR probiotic* OR synbiotic* OR "Lactobacillus" OR lactobacill* OR lactobacteri* OR "Bifidobacterium" OR bifidobacter* OR "Lactococcus" OR lactococc* OR saccharomyce* OR "Saccharomyces" OR streptococcus thermophilus OR "lactic acid bacteri*" OR "bacillus subtilis" OR "enterococcus faecalis" OR yoghurt OR yoghourt OR yogurt OR "Fecal Microbiota Transplantation" OR FMT OR ((feces OR faeces OR fecal) NEAR/3 (infus* OR transplant* OR transfer* OR enema* OR donor* OR transfusion*)) OR "fecal bacteriotherap*" OR "faecal bacteriotherap*") |
| PubMed | ("Child Development Disorders, Pervasive"[MeSH Terms] OR "Speech Disorders"[MeSH Terms] OR autis* OR ASD OR pervasive developmental disorder* OR kanner* OR (language NEAR/4 (delay* OR disabilit* OR disorder* OR impair*)) OR ((communicat* OR speech) NEAR/4 disorder*) OR asperg* NOT aspergill*) AND (microbiota* OR microbiome* OR "Gastrointestinal Microbiome"[MeSH Terms] OR microbiotica OR metagenome* OR ((gut OR gastrointestinal OR intestin*) OR (flora OR microflora OR microbiota)) OR prebiotic* OR probiotic* OR synbiotic* OR "Lactobacillus"[MeSH Terms] OR lactobacill* OR lactobacteri* OR "Bifidobacterium"[MeSH Terms] OR bifidobacter* OR "Lactococcus"[MeSH Terms] OR lactococc* OR saccharomyce* OR "Saccharomyces"[MeSH Terms] OR streptococcus thermophilus OR lactic acid bacteri* OR bacillus subtilis OR yoghurt OR yoghourt OR yogurt OR "Fecal Microbiota Transplantation"[MeSH Terms] OR FMT OR ((feces OR fecal OR microbiota OR stool OR microbiome* OR microflor*) NEAR/3 (infus* OR transplant* OR transfer* OR enema* OR donor* OR transfusion*)) OR fecal bacteriotherap*) NOT (REVIEW[Publication Type]) NOT (SYSTEMATIC REVIEW[Publication Type]) NOT (META-ANALYSIS[Publication Type]) NOT (CASE REPORTS[Publication Type]) NOT (LETTER[Publication Type]) NOT (COMMENT[Publication Type]) AND (humans[Filter]) AND (english[Filter]) |

**Table S2**: Characteristics of included studies

| Study | Country | Study design | Sample size n (male) | Mean age (years, SD) | Intervention | Dose | Comparison | Duration of intervention | Behavioral Findings | Gastrointestinal Findings |
| --- | --- | --- | --- | --- | --- | --- | --- | --- | --- | --- |
| **Probiotics** |  |  |  |  |  |  |  |  |  |  |
| (Arnold et al., 2019) | USA | RCT, double-blind, crossover | 10 (6) | Probiotic, then placebo: 8.83 (2.80)  Placebo, then probiotic: 8.76 (1.18) | De Simone Formulation (Visbiome®) (4  strains of Lactobacilli [*L. casei*, *Lp. plantarum*, *L.*  *acidophilus*, and *L. delbrueckii* subsp.  *bulgaricus*], 3  strains of  *Bifidobacteria* [*B. longum* subsp. *longum*, *B. longum* subsp. *infantis*, and *B.*  *breve*], one strain of *S. thermophilus*,  and starch) | 900 billion  CFU/packet  (1 packet/day) | Matched placebo used; composition not reported | 8 weeks, 3 weeks washout, then another 8 weeks | No change in PRAS-ASD, ABC, PSI, SRS, CSHQ (compared with placebo) | No change in PedsQL GI Total |
| (Lin et al., 2024) | China | RCT, double-blind, parallel | 60 (53) | 2.3-10^*^ | *Bacteroides fragilis* BF839 | ≥1 million CFU/bar  (2 bars/day) | Maltodextrin (10g/bar) | 16 weeks | Reduction in AuBC (body and object use)  No change in AuBC total score, CARS, SRS, S-M standardized score  (compared with placebo) | No change in GSRS |
| (Liu et al., 2019) | Taiwan | RCT, double-blind, parallel | 80 (80) | 10 (2.32) | *Lp. plantarum* PS128 | 30 billion CFU/capsule  (2 capsules/day) | Microcrystalline cellulose | 4 weeks | No change in AuBC-Taiwan version, SRS-Taiwan version, CBCL, CGI-S and CGI-I, SNAP-IV-Taiwan version  (compared with placebo) | N/A |
| (Y.-W.Liu et al., 2023) | Taiwan | RCT, double-blind, parallel | 82 (72) | Early group:  4.99 (1.16)  Late group:  4.66 (1.17) | *Lp. plantarum* PS128 | 30 billion CFU/capsule  (2 capsules/day) | Microcrystalline cellulose | 4 months | Reduction in ASEBA (anxious/depressed), No change in ADHDT  (compared with placebo) | N/A |
| (Rojo‑Marticella et al., 2025) | Spain | RCT, double-blind, parallel | 42 (35) | 9.6 (3.1) | *Lactiplantibacillus* mixture, containing two strains:  *Lp. plantarum*,  *L. brevis* | 1 billion CFU/sachet  (1 sachet/day) | Maltodextrin | 3 months | No change in SRS (compared with placebo) | N/A |
| (Li et al., 2024a) | China | Open-label, single-arm | 72 (NR) | 3-12^*^ | Probio-M8 powder (*Bifidobacterium animalis* subsp. *lactis*) | 50 billion CFU/g  (2g/day) | N/A | 3 months | Reduction in CARS  (relative to baseline) | Reduction in GSRS |
| (Shaaban et al., 2018) | Egypt | Open-label, single-arm | 30 (19) | 7.06 (1.36) | Powder-containing dried carrots with 3 bacterial strains: *L. acidophilus*, *Lc. rhamnosus*, and *B. longum* subsp*. longum* | 100 million CFU/g  (5g/day) | N/A | 3 months | Reduction in Total and subdomain ATEC  (relative to baseline) | Reduction in Total 6-GSI and subdomains constipation, stool consistency, flatulence, and abdominal pain |
| (West et al., 2013) | USA | Open-label, single-arm | 33 (NR) | 7.92 (NR) | Delpro® (*L. acidophilus*, *Lc. casei*, *L. delbrueckii*, *B. longum* subsp*. longum*, *B. bifidum*;  Del-immune V® powder (Lysed, lyophilized powder from *Lc. rhamnosus V* [contains peptidoglycan, muramyl peptides, and DNA motifs]) | Delpro®:  10 billion CFU/capsule  (3 capsules/day)  Del-immune V® powder:  8 mg/day | N/A | 3 weeks | Reduction in Total and subdomain ATEC scores (relative to baseline) | No change in 21-day stool  frequency diary |
| (Kong et el., 2021) | USA | Pilot RCT, double-blind, parallel | 35 (26) | 3-20^*^ | *Lp. plantarum* PS128 (single strand probiotic), oxytocin nasal spray | 30 billion CFU/capsule  (2 capsules/day) | Microcrystalline cellulose capsules | 28 weeks, at week 16 both groups received OXT | Reduction in CGI-I (in OXT + PS128)  No change in ABC, SRS, CGI-S (compared with placebo) | No change in GSI |
| (Meguid et al., 2022) | Egypt | Open-label, single-arm | 40 (27) | 2-5^*^ | *Bifidobacterium* spp. and *Lactobacillus* spp. | 100 million CFU/g  (10g/day) | N/A | 3 months | Reduction in CARS (relative to baseline) | N/A |
| (Mensi et al., 2021) | Italy | Retrospective observational | 131 (122) | 7.18 (3.43) | *Lp. plantarum* PS128 | <30 kg weight: 30 billion CFU/day  ≥30 kg weight: 60 billion CFU/day | N/A | 6 months | Reduction in CGI-I (relative to baseline) | N/A |
| (Niu et al., 2019) | China | Open-label, two-arm | 114 (95) | 4.50 (N/A) | Probiotic mixture powder (*L. bulgaricus* LB42, *L. acidophilus* LA85, *Lc. casei* LC89, *B.longum* subsp*. infantis* BI45, *B. longum* subsp*. longum* BL21, *B. bifidum* Bbi32) + ABA training | 6 billion CFU/g (6g/day) | ABA training | 4 weeks | Reduction in Total and subdomain ATEC score (relative to baseline) | Reduction in GI abnormality score in both children with and without preexisting GI problems (unspecified questionnaire) |
| (Santocchi et al., 2020) | Italy | RCT, double-blind, parallel | 85 (71) | 4.15 (1.08) | De Simone Formulation (Visbiome®) (8 probiotic strains: *S. thermophilus*, *B. breve*, *B. longum longum* subsp*. longum*, *B. longum* supbsp. *infantis*, *L. acidophilus*, *Lp. plantarum*, *Lc. paracasei*, *L. delbrueckii* subsp. bulgaricus) | 450 billion CFU/packet (2 packets/day in the first month and 1 packet/day in the following 5 months) | Maltose and silicon dioxide | 6 months | No change in ADOS-CSS, ADI-R, SCQ, RBS- Revised, VABS-II, CBCL (compared with placebo) | No change in Total GSI, Total 6-GSI  Reduction in Total 6-GSI, GSI- stool smell in subjects with preexisting GI problems |
| (Guidetti et al., 2022) | Italy | RCT, double-blind, crossover | 61 (11) | 2-16 * | *L. fermentum* LF10, *L. salivarius* LS03, *Lp. plantarum* LP01, *B. longum* subsp. *longum* (DLBL07-11) | 10 billion CFU/sachet (2 sachets/day for the first month, then 1 sachet/day for 2 months) | 2.5 g of maltodextrin | 8 months (8 weeks washout period) | Reduction in Total PSI  Increase in VABS, PEP3 (compared to placebo) | Reduction in GSI (Abdominal pain, diarrhea symptoms) |
| (Mazzone et al., 2023) | Italy | Pilot RCT, double-blind, parallel | 43 (35) | Intervention: 6.23 (1.15)  Control: 5.94 (1.29) | BioGaia Gastrus (*Li.* *reuteri* which contains  DSM 17938 and ATCC PTA 6475 | ≥ 200 million CFU/tablet  (2 tablets/day) | Matched placebo used; composition not reported | 6 months | Reduction in SRS (total and social communication), ABAS-2 (only in social adaptive composite score)  No change in ADOS-2, RBS-R, CBCL, PSI/SF  (compared with placebo) | No change in GSRS |
| **Prebiotics** |  |  |  |  |  |  |  |  |  |  |
| (Grimaldi et al., 2018) | UK | RCT, double-blind, parallel | 41 (31) | 4-11^*^ | Exclusion diet + B- GOS® mixture  (Bimuno®, a galactooligosaccharide)  or Un-restricted  diet + B-GOS®  mixture | 1.8 g  (80% GOS content) | Exclusion/Un-restricted diet + Maltodextrin—  GLUCIDEX® | 6 weeks | Reduction in ATEC (anti-social scale), AQ (social skills)  No change in EQ-SQ, SCAS-P (exclusion diet +  probiotics  compared to other  three groups) | No change in Abdominal pain,  bowel  movement,  bloating,  flatulence, and  stool form  (unspecified  questionnaire) |
| (Palmer et al., 2024) | Australia | Pilot RCT, double-blind, parallel | 41 (27) | 7.18 (1.36) | GOSYAN® (GOS) | 1.2 g/capsule (2 capsules/day for the first week, then 4 capsules/day for the final 5) | Maltodextrin D28, Manildra®, 2.4 g/day | 6 weeks | No change in SRS-2, BAMBI, QoLA part A and B (compared with placebo) | No change in Total 6-GSI scores |
| (Raghavan et al., 2022) | Japan | Pilot RCT, open-label, parallel | 18 (NR) | NR | Nichi Glucan (black yeast-derived AFO-202 beta-glucan) + L-Carnosine 500 mg/day | 0.5 g/sachet (2 sachets/day) | L-Carnosine 500 mg/day | 3 months | Reduction in CARS (compared with control) | N/A |
| (Inoue et al., 2019) | Japan | Open-label, single-arm | 13 (12) | 5.9 (2.2) | Partially hydrolyzed guar gum | 6 g/day | N/A | 2–15 months (median = 2) | Reduction in ABC-Irritability (relative to baseline) | Increase in Frequency of defecation (unspecified questionnaire) |
| **Synbiotics** |  |  |  |  |  |  |  |  |  |  |
| (Wang et al., 2020) | China | RCT, double-blind, parallel | 26 (24) | 4.3 (NR) | *B. longum* subsp*. Infantis,* Bi-26, *Lc. rhamnosus* HN001, *B. animalis* subsp. *lactis* BL-04, *Lc. paracasei* LPC-37 + FOS | 10 billion CFU/pack (1 pack/day) | Maltodextrin | 30, 60, or 108 days | Reduction in Total ATEC score and subdomains: speech/language/communication and sociability  (relative to baseline) | Reduction in Total 6-GSI score and subdomains constipation, diarrhea, and stool smell |
| (Phan et al., 2024) | China | Pilot open-label, single-arm | 296 (236) | 10.41 (NR) | Synbiotics (content NR) | NR | N/A | 3 months | No change in SRS (relative to baseline) | Reduction in GSRS |
| (Mitchell et al., 2024) | Australia | Open-label, parallel | 31 (18) | 7.75 (1.87) | Synbiotics (content NR) | NR | N/A | 12 weeks | Significant reduction in ABC- Stereotypic Behaviour  (relative to baseline) | Reduction in total 6-GSI, and subdomains including pain and stool smell |
| (Sanctuary et al., 2019) | USA | Pilot RCT, double-blind, crossover | 8 (7) | 6.8 (2.4) | Probiotic (*B. longum* subsp*.* *infantis*) + BCP (oligosaccharides) and other nutrients (i.e., calcium, sodium, and potassium); 0.15 g/lb (body weight)/day | 20 billion CFU/day | BCP | 5 weeks, 2 weeks washout, then another 5 weeks | No change in Total ABC, RBS-R, ABAS-II reduction in ABC-Stereotype (Combination vs. BCP Only) | No change in QPGS-RIII, GIH |
| (Schmitt et al., 2023) | USA | RCT, double-blind, crossover | 15(15) | 20.0 (3.05) | Oral SB-121 (a combination of *Li. reuteri*, Sephadex® (dextran microparticles; 200mg), and maltose) | 20 billion CFU/day | Sephadex® and maltose | 28 days SB-121 or placebo followed by 14-day washout then 28 days of dosing with other treatment | No change inVineland-3, ABC, CGI-I and CGI-S (compared with placebo) | N/A |
| **FMT** |  |  |  |  |  |  |  |  |  |  |
| (Chen et al., 2024) | China | Open-label, single-arm | 29 (NR) | 2-11^*^ | Two oral administrations daily, each with 2 FMT capsules, for 12 consecutive days | The aggregated monthly administration of 48 capsules contained bacteria equivalent to 200g fresh stool | N/A | 4 months | Reduction in AuBC, CARS  (relative to baseline) | Reduction in GSRS, Increase in BSFS |
| (Wang et al., 2024) | China | RCT, double-blind, parallel | 41 (38) | 6.54 (NR) | FMT was administered orally during the first and fifth weeks, followed by an additional four weeks of observation | NR | Matched placebo used; composition not reported | 5 weeks | Reduction in ABC, CARS, SRS  (compared with placebo) | Reduction in GSRS |
| (Li et al., 2021) | China | Open-label, single arm | 40 (37) | 8.03 (3.73) | Freeze-dried FMT capsules or colonoscopic FMT (10 g/50 kg/child). | 200 trillion CFU/week | N/A | 4 weeks | Reduction in ABC, CARS, SAS, SRS  (relative to baseline) | Reduction in GSRS  Increase in BSFS |
| (N.-H. Liu et al., 2023) | China | Open-label, single-arm | WMT1: 24  WMT2: 18  WMT3: 13  WMT4: 8 | NR | WMT via TET | NR | N/A | NR | WMT1:  Reduction in ABC, SDSC  WMT2:  Reduction in ABC, SDSC  WMT3:  Reduction in ABC, SDSC  WMT4:  No change in ABC,  Reduction in SDSC  (relative to baseline) | Increase in BSFS in all courses |
| (Li et al., 2024b) | China | Open-label, single arm | 38 (32) | 2-17^*^ | Oral lyophilized FMT; each course was completed within 3 days, with one course every 4 weeks for a total of 3 courses over 12 weeks | 1 g of donor stool per 1 kg of recipient body weight | N/A | 12 weeks | Reduction in AuBC, CARS, SRS, SDSC  (relative to baseline) | Reduction in GSRS |
| (Li et al., 2024c) | China | Open-label, single arm | 98 (80) | 7 (NR) | Three FMT courses over 12 weeks, using capsules or fecal solution. Capsules are taken before meals for 3 days, repeated every 4 weeks. Fecal solution was administered via a tube for 3 days, repeated every 4 weeks. | NR | N/A | 8 weeks | Reduction in AuBC, CARS, SRS  (relative to baseline) | Reduction in GSRS |
| (Pan et al., 2022) | China | Retrospective observational | 42 (34) | 6 (NR) | WMT. The fecal suspension was administered via a TET (60–90 mL/day for 6 consecutive days) | About 50 trillion bacteria | N/A | NR | Reduction in ABC, SDSC and CARS (relative to baseline) | Reduction in constipation (unspecified questionnaires or qualitative GI diary) |
| (Kang et al., 2017, 2019) | USA | Open-label, single- arm | 18 (16) | 10.8 (1.6) | SHGM. Oral group: given over 2 days (divided into 3 doses/day). Rectal group: single administration over 1 hour. | Initial dose: 2.5 trillion cells/day and maintenance dose: 2.5 billion cells/day for 7 or 8 weeks | N/A | 10 weeks, further follow-up at week 18 and at year 2 | Reduction in PGI-III, CARS, SRS, VABS, ABC (relative to baseline) | Reduction in GSRS, Daily Stool Record |
| (Zhang et al., 2022) | China | Retrospective observational | 49 (41) | Constipation group:  5.67 (3.08)  Control group: 6.72 (3.94) | WMT through TET or nasojejunal tube + probiotics before and during WMT for 2 courses (W1 and W2) | 120 mL administered daily for 6 days per course | N/A | NR | Constipation group:  Reduction in CARS, AuBC, SDSC (after W2) Control group:  Reduction in CARS (after W1 and W2)  No change in ABC and SDSC  (relative to baseline) | Constipation group:  Increase in BSFS (after W1 and W2) Control group:  No change in BSFS |

Abbreviations: **ABA** - Applied Behavior Analysis; **ABAS-2** - Adaptive Behavior Assessment System, Second Edition; **ABC** - Autistic Behavior Checklist; **ADHDT** - Attention-deficit/hyperactivity Disorder Test; **ADOS** - Autism Diagnostic Observation Schedule; **ADOS-CSS** - Total ADOS calibrated severity score; **ASEBA** - Achenbach System of Empirically Based Assessment; **ATEC** - Autism treatment evaluation checklist; **AuBC** – Autism Behavior Checklist; **BAMBI** - Brief Autism Mealtime Behavior Inventory; **BCP** - Bovine colostrum product; **BSFS** – Bristol Stool Form Scale; **CARS** - Childhood Autism Rating Scale; **CBCL** - Child Behavior Checklist; **CFU** - Colony-forming units; **CGI** - Clinical Global Impression; **CGI-I** - Clinical global impression-Improvement; **CGI-S** - Clinical global impression-Severity; **CSHQ** - Children's Sleep Habits Questionnaire; **DQ** - Developmental Quotients; **DSM** - Diagnostic and Statistical Manual of Mental Disorders; **EQ-SQ** - Empathy/systemizing Quotient; **FMT** - Fecal microbiota transplant; **FOS** - Fructo-oligosaccharides; **GI** - Gastrointestinal; **GDH** - gut-directed hypnotherapy; **GIH** - Gastrointestinal History survey; **GOS** - Galacto-oligosaccharides; **GSI** - Gastrointestinal severity index; **GSRS** - Gastrointestinal symptom rating scale; **OXT** – Oxytocin; **PedsQL** - Pediatric Quality of Life Inventory; **PEP3** - Psycho educational profile 3rd edition; **PGI-III** - Parent global impressions-III; **PRAS-ASD** - Parent-Rated Anxiety Scale for Youth With Autism Spectrum Disorder; **PSI** - Parenting Stress Index; **PSI/SF** - Parenting Stress Index Short Form; **QoLA** - Quality of life in autism Questionnaire; **QPGS-RIII** - Questionnaire on Pediatric Gastrointestinal Symptoms: Rome III version; **RBS-R** - Repetitive Behaviors Scale-Revised; **RCT** – Randomized controlled trial; **SAS** – Self-rating anxiety scale; **SCAS-P** - Spence children’s anxiety scale-parent version; **SDSC** - Sleep Disturbance Scale for Children; **SHGM** - Standardized Human Gut Microbiota; **S-M** - Normal Development of Social Skills from Infants to Junior High School Children; **SNAP–IV** - Swanson, Nolan, and Pelham rating scale version 4; **SRS** - Social Responsiveness Scale; **SRS-2** – Social Responsiveness Scale. 2^nd^ edition; **TET** - Transendoscopic Enteral Tube; **UK** - United Kingdom; **USA** - United States of America; **VABS** - Vineland Adaptive Behavior Scale; **VABS-II** - Vineland Adaptive Behavior Scales-II; **W1** and **W2** - Wash 1 and 2; **WMT** - washed microbiota transplant.

^*^: Mean and standard deviation were not reported, only ranges were available.

**Table S3**: Results of the quality assessment for non-randomized clinical trials, using the Methodological index for non-randomized studies (MINORS)

| Study ID | Methodological index for non-randomized studies (MINORS) | | | | | | | | | | | | | | |
| --- | --- | --- | --- | --- | --- | --- | --- | --- | --- | --- | --- | --- | --- | --- | --- |
|  | Criteria for all studies | | | | | | | | | Additional criteria for comparative studies | | | | | Overall study quality |
|  | A | B | C | D | E | F | G | H | Score (out of 16) | I | J | K | L | Score (out of 24) |  |
| Niu, 2019 | 2 | 2 | 2 | 2 | 2 | 2 | 2 | 0 |  | 1 | 0 | 2 | 2 | 19 | High quality |
| Mitchell, 2024 | 2 | 2 | 2 | 2 | 1 | 0 | 2 | 2 |  | 2 | 0 | 0 | 2 | 17 | Fair |
| N.-H. Liu, 2023 | 2 | 2 | 1 | 2 | 0 | 0 | 0 | 0 | 7 |  |  |  |  |  | Poor |
| Meguid, 2022 | 2 | 2 | 2 | 2 | 2 | 2 | 2 | 0 | 14 |  |  |  |  |  | High quality |
| Inoue, 2019 | 2 | 1 | 2 | 2 | 0 | 0 | 0 | 0 | 7 |  |  |  |  |  | Poor |
| Kang, 2017, 2019 | 2 | 2 | 2 | 2 | 0 | 2 | 2 | 0 | 12 |  |  |  |  |  | Fair |
| Phan, 2024 | 2 | 2 | 0 | 1 | 0 | 1 | 1 | 0 | 7 |  |  |  |  |  | Poor |
| Shaaban, 2018 | 2 | 0 | 2 | 0 | 1 | 2 | 2 | 0 | 8 |  |  |  |  |  | Poor |
| Li, 2021 | 2 | 2 | 1 | 1 | 0 | 2 | 2 | 0 | 10 |  |  |  |  |  | Fair |
| Li, 2024a | 2 | 0 | 1 | 2 | 1 | 0 | 1 | 0 | 8 |  |  |  |  |  | Poor |
| Li, 2024b | 2 | 1 | 2 | 2 | 2 | 0 | 1 | 0 | 10 |  |  |  |  |  | Fair |
| Li, 2024c | 2 | 2 | 2 | 2 | 2 | 2 | 2 | 2 | 16 |  |  |  |  |  | High quality |
| Chen, 2024 | 2 | 0 | 2 | 2 | 2 | 1 | 2 | 0 | 11 |  |  |  |  |  | Fair |
| West, 2013 | 0 | 0 | 1 | 2 | 0 | 0 | 0 | 1 | 4 |  |  |  |  |  | Poor |

Criteria (A-L): (A) A clearly stated aim; (B) Inclusion of consecutive patients; (C) Prospective collection of data (D) Endpoints are appropriate to the aim of the study; (E) Unbiased assessment of the study endpoint; (F) Follow-up period appropriate to the aim of the study; (G) Loss to follow-up less than 5%; (H) Prospective calculation of the study size; (I) An adequate control group; (J) Contemporary groups; (K) Baseline equivalence of groups; (L) Adequate statistical analysis.

**Table S4**: Results of the quality assessment of the retrospective observational studies without controls, using the Modified Newcastle-Ottowa scale for cohort studies

| Study ID | Modified Newcastle-Ottowa scale for cohort studies | | | | | | | |
| --- | --- | --- | --- | --- | --- | --- | --- | --- |
|  | Selection | | | Outcome | | | Score (out of 6) | Overall study quality |
|  | Representativeness of the exposed cohort (*) | Ascertainment of exposure (*) | Demonstration that outcome of interest was not present at start of study (*) | Assessment of outcome (*) | Was follow-up long enough for outcomes to occur (*) | Adequacy of follow-up of cohorts (*) |  |  |
| Pan, 2022 | * | * | * | * | - | * | 5 | High quality |
| Mensi, 2021 | * | * | * | * | * | * | 6 | High quality |
| Zhang, 2022 | - | * | * | * | * | - | 4 | Fair |
